# Silencing of CA1 mRNA in tumour cells does not change the gene expression of the extracellular matrix proteins

**DOI:** 10.1111/jcmm.13315

**Published:** 2017-08-07

**Authors:** Radivojka Vulic, Silvia Tyciakova, Maria Dubrovcakova, Ludovit Skultety, Jan Lakota

**Affiliations:** ^1^ Institute of Virology BMC SAS Bratislava Slovakia; ^2^ Cancer Research Institute BMC SAS Bratislava Slovakia; ^3^ St. Elizabeth Cancer Institute Bratislava Slovakia; ^4^ Institute of Normal and Pathological Physiology SAS Bratislava Slovakia

**Keywords:** siCA1, siMock, carbonic anhydrase I, tumour cells, COL1A1, COL4A4, CTHRC1, LAMC2, WNT7B

## Abstract

We report the silencing of CA1 mRNA in PC3 and MDA cells. The levels of mRNA coding CA1 protein in the knock‐down mRNA (CA1 siRNA) cells have been measured by RT‐PCR and were approximately 5% (PC3) and 20% (MDA‐MB‐231), respectively, of the level of control (Mock siRNA) used during silencing. In PC3 and MDA‐MB‐231 cells, the mRNAs for COL1A1 and COL4A4 were up‐regulated. The mRNAs for CTHRC1, LAMC2, and WNT7B were not changed when compared to the control. The morphology of the cells during the treatments remained the same. On the Western blots, the lysate from the silenced cells showed lower levels of CA I as well.

## Introduction

Carbonic anhydrases (CAs) are zinc metalloenzymes that catalyse the reversible hydration of carbon dioxide to a bicarbonate ion and proton: H_2_O + CO_2_↔HCO_3_^−^ + H^+^ 1, 2. Besides being ubiquitous in all life forms, it was found that the CA family performs numerous functions in many different organisms. CAs are both a drug target, with their inhibitors having applications ranging from diuretics, antiglaucoma agents, epilepsy, obesity, antitumour diagnostic tools/drugs, to catalysts for biotechnological application in CO_2_ capture and other processes.

The first isoform of CAs, CA I, was first discovered in vertebrate erythrocytes 3. It is also found in the kidneys, colon, lungs, brain, and eyes 4. Besides a role in the process of pH homoeostasis, respiration, and in erythroid differentiation, CA I can be involved in some pathological processes such as anaemia, chronical acidosis, diabetic macular oedema, proliferative diabetic retinopathy, and vasogenic oedema 5, 6. The expression of CA I has been investigated in various types of malignancies. The low level of CA I in the colonic epithelial cells might serve as an indicative and specific marker for prediction of colorectal cancer 7. On the other hand, Takakura *et al*. 8 showed that patients with prostate cancer contained an increased level of CA I peptide fragments in the plasma compared to the plasma of healthy controls. The CA I protein overexpression is probably strictly associated with increased CA I production and secretion in prostate cancer cells. Thus, the elevated level of CA I protein in plasma might represent a potential biomarker for prostate cancer.

Interestingly, in our previous work we reported that some patients who relapsed after high‐dose therapy (HDT) and autologous stem cell transplantation (ASCT) were positive for autoantibodies against CA I, and the tumours in these patients spontaneously regressed 9. Thus, these autoantibodies were suggested as markers of good prognosis. These patients’ sera, which were positive for autoantibodies against CA I, were further used to examine their effect on the biology of tumour cells grown *in vitro*. We have shown that the expression of the CA1 mRNA was up‐regulated in the tumour cells during the treatment of the above‐mentioned patients’ sera. The mRNAs coding for proteins associated with basal lamina assembly, cytoskeleton, WNT7B and collagen triple helix repeat containing 1 (CTHRC1) were down‐regulated 10. To examine the effect of the opposite phenomenon, the CA1 mRNA was silenced by RNA interference system in PC3 and MDA‐MB‐231 cells. We show that knock‐down of the CA1 mRNA in tumour cells enhances/does not change the gene expression of the extracellular matrix (ECM) proteins.

## Materials and methods

### Cell lines and chemicals

A human prostate adenocarcinoma cell line derived from metastatic site PC3 (ATCC^®^CRL‐1435TM) and a human breast adenocarcinoma cell line MDA‐MB‐231 (ECACC 92020424) were maintained in a high‐glucose (4.5 mg/ml) DMEM (Biochrom AG, Berlin, Germany) supplemented with 10% foetal calf serum (FCS) (Lonza BioWhittaker, Switzerland) and gentamicin (Sandoz, Munich, Germany) at 37°C in humidified air with 5% CO_2_. All chemicals were purchased from Sigma‐Aldrich (St Louis, MO, USA) unless stated otherwise.

### CA1 silencing

For the transient silencing of the CA1 gene, PC3 and MDA‐MB‐231 cells were seeded (3 × 10^6^ cells) in a 10‐cm Petri dish. After 24 hrs, the cells were transfected with siCA1 to silence CA I expression and with siMock as a control using DharmaFECT^™^ (GE Healthcare, Chicago, IL, USA.) according to the manufacturer's recommendations. The next day, the medium was replaced and the cells were incubated for 48 hrs. Two days later, the cells were collected and frozen for reverse transcriptase quantitative PCR analyses or lysed in a RIPA buffer and analysed by Western blot.

### Western blotting

The cell lysates were centrifuged (10 min. at 17,000× *g*), and the total protein concentrations in supernatants were determined by BCA assay. Samples of 100 μg total proteins were separated using electrophoresis in the 10% SDS‐PAGE and blotted onto the PVDF membrane (Millipore, Billerica, MA, USA). The membrane was blocked for 2 hrs in a blocking buffer containing 5% non‐fat milk in PBS with 0,1% Tween 20. Incubation with primary antibodies was performed overnight at 4°C. A CA I mouse monoclonal antibody (Moravian Biotechnology, Brno, Czech Republic) was diluted 1:1000 in 3% BSA in TBST. Then, the membrane was washed with 0.1% Tween 20 in PBS, incubated for 1 hr (RT) with secondary antibody (Sigma‐Aldrich), washed again and developed with the ECL detection system.

### Gene expression analyses

Reverse transcriptase quantitative PCR (RT‐qPCR) was performed as previously described 10.

## Results

**Figure 1:**
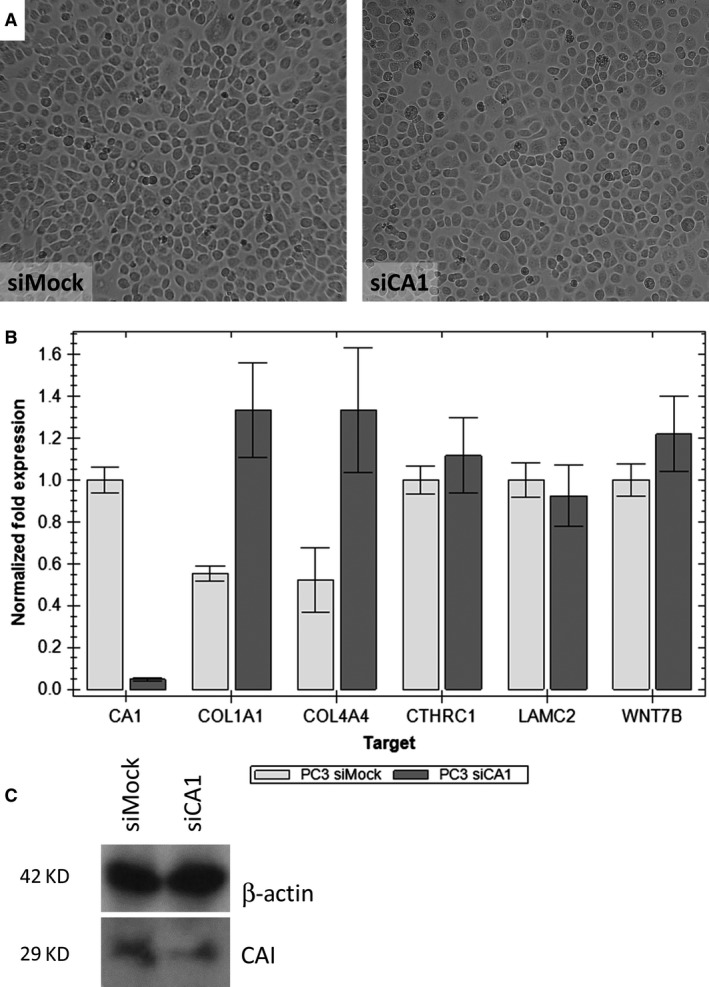
The effect of silencing carbonic anhydrase I (CA I) on the PC3 prostate cancer cell morphology and expression**.** (**A**) A light‐microscope image of PC3 cells 48 hrs after siCA1 transfection compared to PC3 cells transfected with Mock siRNA. Magnification 100x. (**B**) Gene expression analysis of silenced CA1 gene and selected genes responsible for basal lamina assembly (COL1A1—collagen type I alpha 1, COL4A4—collagen type IV alpha 4, LAMC2—laminin subunit gamma 2, CTHRC1—collagen triple helix repeat containing 1, WNT7B—Wingless‐Type MMTV Integration Site Family, Member 7B); reverse transcriptase quantitative PCR (RT‐qPCR). (**C**) Western blot analysis indicating reduced amount of CA I protein after CA1 mRNA silencing. Beta‐actin was used as a loading control.

**Figure 2:**
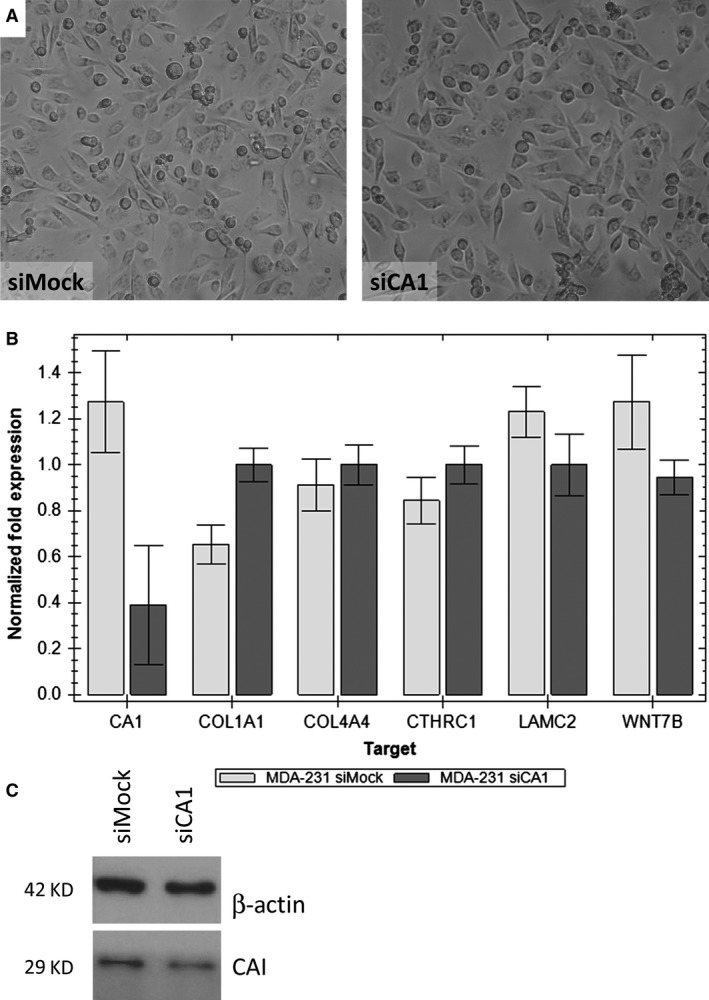
The effect of silencing carbonic anhydrase I (CA I) on the MDA‐MB‐231 breast carcinoma cell morphology and expression. (**A**) A light‐microscope image of MDA‐MB‐231 cells 48 hrs after siCA1 transfection compared to parental MDA‐MB‐231 cells transfected with Mock siRNA. Magnification 200x. (**B**) Gene expression analysis of silenced CA1 gene and selected genes responsible for basal lamina assembly (COL1A1—collagen type I alpha 1, COL4A4—collagen type IV alpha 4, LAMC2–laminin subunit gamma 2, CTHRC1—collagen triple helix repeat containing 1, WNT7B—Wingless‐Type MMTV Integration Site Family, Member 7B); reverse transcriptase quantitative PCR (RT‐qPCR). (**C**) Western blot analysis indicating reduced amount of CA I protein after CA1 mRNA silencing. Beta‐actin was used as a loading control.

First, we compared the morphology of the different cell lines—PC3 (prostatic carcinoma) and MDA‐MB‐231 (breast carcinoma). Tumour cells with silenced CA1 gene (siCA1) compared with their negative control (siMock) did not show any morphological changes (Figs 1A and 2A). The level of transient silencing of the CA1 gene was checked by RT‐qPCR and WB analyses (Figs 1B, C and 2B, C). An extract from cells with silenced CA1 gene showed a lower amount of the enzyme CA I (Figs 1C and 2C). Moreover, the results from the RT‐qPCR confirmed the successful silencing of CA1 gene in both cell lines. The amount of CA1 mRNA (mean Ct 33.26) was approximately 5% of the control/Mock RNA (mean Ct 28.46) after silencing (Fig. 1B) in case of PC3 cells, while for the MDA‐231‐MB cells, the effect of silencing was about 20% of the control/Mock RNA (mean Ct 37.73 *versus* 36.37) (Fig. 2B).

Subsequently, we checked the effect of silencing of the CA1 gene on the expression of the mRNAs for all selected ECM genes: COL1A1, COL4A4, CTHRC1, LAMC2, WNT7B (Figs 1B and 2B). We focus on these genes because in our previous work, we showed that the presence of anti‐CA I autoantibodies changes tumour cell morphology *in vitro* and changes the gene expression profile. All of these genes for proteins of the basal lamina and cytoskeleton were down‐regulated and in agreement with the observed morphological changes 10.

Interestingly, results from RT‐qPCR were opposite to the results mentioned above. PC3 cells with silenced CA1 gene, mRNAs for COL1A1, COL4A4 were up‐regulated while the mRNAs for CTHRC1, LAMC2, and WNT7B were not changed when compared with the control/Mock RNA (Figs 1B and 2B). The same upregulation was also observed in the case of MDA‐231‐MB cells, but was not as significant as in case of PC3 cells.

## Discussion

We have performed the silencing of the CA1 mRNA in the tumour cell lines PC3 and MDA‐MB‐231. We decided to use the PC3 cells because the CA1 mRNA is abundantly present in this line and the MDA‐MB‐231 cells because the CA1 mRNA is present only marginally 10. The silencing of the CA1 mRNA is presented in Figs 1B and 2B, respectively. Both knock‐downs are accompanied with the upregulation of the mRNAs coding the proteins collagen I and IV. The mRNAs coding the proteins CTHRC1, laminin gamma, and WNT7B were approximately the same (Figs 1B and 2B). Moreover, we did not observe any morphological cell changes (Figs 1A and 2A). These data are in strong albeit indirect agreement with the historical paper of Kendall and Tashian 11. There, the authors describe a family from the Greek island Icaria with virtually absent CA I (0.6–0.7 ng CA I/mg haemoglobin in contrast to normal values 11.57 ± 2.26 μg CA I/mg haemoglobin) with no clinical consequences.

When the tumour cells were grown in the presence of the anti‐CA I positive sera of the patients with spontaneous tumour regression, the expression of the CA1 gene has been increased. At the same time, we observed the downregulation of genes coding collagen type I alpha 1, collagen type IV alpha 4, laminin subunit gamma 2, CTHRC1 and WNT7B. This effect was accompanied with morphological changes of the tumour cells grown *in vitro* 10.

The decrease/increase of the CA I enzyme in the red blood cells of the human patients has been observed in patients with hyperthyroidism/hypothyroidism 12. Hypothyroidism in humans is usually associated with anaemia 13. In a recent paper 14, the authors compared the risk of breast cancer in women with hypothyroidism (61,873 women) and hyperthyroidism (80,343 women). Hypothyroidism was associated with a lower risk of breast cancer (SIR: 0.94, 95% CI: 0.88–1.00) when compared to the general population; hyperthyroidism was associated with a higher risk of breast cancer (SIR: 1.11, 95% CI: 1.07–1.16) when compared to the general population 14. The above‐mentioned data can potentially highlight the spontaneous tumour regression like Ariadne's thread in the labyrinth: statistically, the incidence of breast cancer in women with hypothyroidism is lower compared to the general population. The amount of CA I enzyme in the patients with hypothyroidism is statistically higher. Moreover, these patients are anaemic. The patients with spontaneous tumour regression contain anti‐CA I autoantibodies. This is always accompanied by a varying degree of aplastic anaemia (AA)‐like syndrome. The treatment of the tumour cells with these sera enhances the CA1 mRNA expression in these cells. This is accompanied by the downregulation of the ECM proteins and oncogenes CTHRC1 and WNT7B. It seems that the ‘obscure’ enzyme carbonic anhydrase I plays the central role in this process.

## Conflict of interest

The authors confirm that there are no conflict of interests.
